# Molecular and serological survey of lyssaviruses in Croatian bat populations

**DOI:** 10.1186/s12917-018-1592-z

**Published:** 2018-09-06

**Authors:** Ivana Šimić, Ivana Lojkić, Nina Krešić, Florence Cliquet, Evelyne Picard-Meyer, Marine Wasniewski, Anđela Ćukušić, Vida Zrnčić, Tomislav Bedeković

**Affiliations:** 1 Croatian Veterinary Institute, Savska cesta 143, 10000 Zagreb, Croatia; 2 ANSES - Nancy Laboratory for rabies and wildlife, Batiment H CS 40009, 54220 Malzeville, France; 3 Croatian Biospeleological Society, Demetrova 1, 10000 Zagreb, Croatia

**Keywords:** Bat, *Lyssavirus*, European bat lyssavirus-1, Croatia, Antibodies

## Abstract

**Background:**

Rabies is the only known zoonotic disease of bat origin in Europe. The disease is caused by species belonging to the genus *Lyssavirus*. Five Lyssavirus species, i.e., European bat lyssavirus (EBLV)-1, EBLV-2, Bokeloh bat lyssavirus, Lleida bat lyssavirus, and West Caucasian bat virus, have been identified in European bats. More recently, a proposed sixth species, Kotalahti bat lyssavirus, was detected. Thus, in this study, active surveillance was initiated in order to obtain insights into the prevalence of lyssaviruses in Croatian bat populations and to improve our understanding of the public health threat of infected bats.

**Results:**

In total, 455 bats were caught throughout Continental and Mediterranean Croatia. Antibodies were found in 20 of 350 bats (5.71%, 95% confidence interval 3.73–8.66). The majority of seropositive bats were found in Trbušnjak cave (Continental Croatia, Eastern part), and most seropositive bats belonged to *Myotis myotis* (13/20). All oropharyngeal swabs were negative for the presence of *Lyssavirus*.

**Conclusions:**

The presence of lyssaviruses in bat populations was confirmed for the first time in Croatia and Southeastern Europe. The results of this study suggest the need for further comprehensive analyses of lyssaviruses in bats in this part of Europe.

## Background

Rabies is a fatal viral zoonotic disease infecting all warm-blooded mammals, including bats, and is caused by viruses belonging to the genus *Lyssavirus.* The World Health Organization (WHO) reported that 59, 000 human deaths occur annually around the world due to dog-transmitted rabies. In contrast, rabies transmitted from bats causes a small proportion of human cases globally [1]. Currently, 16 *Lyssavirus* species are recognized by the International Committee on the Taxonomy of Viruses [2], all of which have been reported in bats except for two species, Mokola lyssavirus and Ikoma lyssavirus [3, 4]. Recently, two related viruses, i.e., Taiwan bat lyssavirus (TWBLV) and Kotalahti bat lyssavirus (KBLV), were isolated from bats [2, 5, 6].

During the last century, analysis of lyssaviruses in bats has shown that bats play an important role as a reservoir for these viruses. In the Americas (New World), only variants of classical Rabies virus (RABV) are associated with bats, whereas across Africa, Asia, Europe (Old World), and Australia no detection of RABV has been reported in any bat species. However, other lyssaviruses have been detected. The long-term association of lyssaviruses with bats suggests that lyssaviruses are the most important and only confirmed zoonotic pathogen of bat origin in Europe [3, 7].

Lyssaviruses are divided into three phylogroups, among which only phylogroup I viruses are all neutralized by existing rabies vaccines [3]. Rabies in European bat populations is caused by five species and two phylogroups: European bat lyssavirus (EBLV) -1 (phylogroup I), EBLV-2 (phylogroup I), Bokeloh bat lyssavirus (BBLV; phylogroup I), West Caucasian bat lyssavirus (WCBV; phylogroup III), and Lleida bat lyssavirus (LLEBV; confirmed phylogroup III) [7]. Recently, a putative species of KBLV (tentatively phylogroup I) was detected in Finland in *Myotis brandtii* [6].

EBLV-1 and EBLV-2 are the two main lyssavirus species detected in bats in Europe. EBLV-1 is detected in the majority of bat rabies cases and is primarily found in serotine bats (*Eptesicus serotinus*), whereas less than 40 EBLV-2 cases have been recorded in Daubenton’s bats (*Myotis daubentonii*) and pond bats (*Myotis dasycneme*) [3, 8]. Few cases of transmissions of EBLV-1 to other terrestrial animals (sheep, stone marten, and domestic cats) and humans have been recorded, confirming that the risk of spillover infection remains low but not negligible [3]. Therefore, additional studies are clearly needed to investigate the distribution and genetic characteristics of lyssaviruses across Europe.

In Europe, bat rabies surveillance is highly heterogeneous in terms of the existing networks of bat biologists, active and passive surveillance, number of bat species submitted for rabies diagnosis and individuals sampled [9]. The passive surveillance is based on the testing of sick bats (bats showing clinical signs or abnormal behaviors linked to rabies) or bats found dead. Active surveillance is based on the monitoring of free-living indigenous bat populations for *Lyssavirus* infections [10]. Some European bat species have never been tested for rabies; thus, their role in the epidemiology of lyssaviruses remains uncertain [9].

The network between bat biologists and rabies scientists in Croatia has been poor and inconsistent, and the number of bats included in passive surveillance was negligible, with only 124 bats submitted for rabies diagnosis from 2010 to 2017. There are 34 insectivorous bat species in Croatia, of which five migrate longer distances [11, 12]. The geographical distribution of each bat species in Croatia is still not clearly defined, despite the efforts of bat biologists. Accordingly, data on bat rabies in Croatia is scarce and not up to date. Initial research on bats and their zoonotic diseases was performed in 1968 for military purposes. The objective was to determine the risk of exposure to zoonotic pathogens in caves, since such underground sites had important roles as hiding places, hospitals, and weapon stores owing to their inaccessibility, constant temperature, and access to water. In these studies, 470 cave-dwelling bats belonging to 11 species (*Myotis myotis*, *Myotis oxygnatus*, *Rhinolophu. blasii*, *R. ferrumequinum*, *R. hipposideros* minimus and hipposideros, *R. euryale*, *Myotis emarginatus*, *Miniopterus schreibersii*, *Pipistrellus kuhlii,* and *R. mehelyi*) were sampled in 15 caves across Croatia. All collected samples were found negative for rabies by laboratory analysis in Prague by using immunofluorescence on inoculated mouse brain [13]. In 1986, the Croatian Veterinary Institute started a study on bat rabies in Croatia and tested around 30 *Eptesicus serotinus* bats, all of which were found negative for rabies by fluorescence antibody test (FAT). These investigations were stopped because of the Croatian War of Independence. Additionally, between 2008 and 2012, 203 dead bats from six genera (*Miniopterus, Myotis, Nyctalus, Rhinolophus, Pipistrellus, Plecotus, Eptesicus,* and *Hypsugo*) collected on various locations around Croatia during field research for inventory purposes were sampled. All samples were found negative by FAT [14]. In this study, we performed active surveillance to investigate the prevalence of EBLVs in bats across Croatia by detecting EBLV-1 antibodies in blood samples using a modified fluorescent antibody virus neutralization (mFAVN) test. The presence of the *Lyssavirus* genome in oropharyngeal swabs of the tested animals was assessed by reverse transcription polymerase chain reaction (RT-PCR). The main objective of this study was to obtain data on the prevalence of lyssaviruses in apparently healthy bats in Croatia in order to improve our understanding of virus distribution and the public health risk associated with bats in Southeastern Europe (SEE).

## Results

In total, 455 bats were caught between 2016 and 2017 (Table 1). Of these bats, 440 bats from seven species (*E. serotinus, Myotis blythii, Myotis emarginatus, Myotis myotis, Myotis nattereri, Miniopterus schreibersii,* and *R. ferrumequinum*) were captured. Fourteen bats were unable to be confidently categorized between *Myotis myotis* and *Myotis blythii* and were therefore designated as *Myotis myotis/blythii*. For one individual, neither species nor sex was determined because the animal escaped. All animals caught in the spring in both years were adults, and only 10 animals caught in autumn of 2017 were subadult. Females (*n* = 241) outnumber males (*n* = 213; Tables 1 and 2.). Most of the trapped bats were *Miniopterus schreibersii* (*n* = 255), followed by *R. ferrumequinum* (*n* = 90) and *Myotis myotis* (*n* = 56). Only one *E. serotinus* and one *Myotis nattereri* were caught (Table 2).

**Table 1:** Number of bats caught through active surveillance

Sex	Spring 2016	Spring 2017	Autumn 2017
Male	56	27	130
Female	120	74	47
Not determined	–	–	1
Total	176	101	178

**Table 2:** Number of bats tested for virus- neutralizing antibodies per species and percentage of positive bats with confidence intervals (CIs) during active surveillance in 2016 and 2017

Species	Number of sampled bats	M	F	Analyzed blood samples	M	F	Obtained results		
							Overall (%pos) [CI]	M (%pos) [CI]	F (%pos) [CI]
ES	1	1	/	1	1	/	1 (0.00)	1 (0.00)	/
MS	222	109	113	210	100	110	200 (2.50) [1.07–5.72]	96 (2.08) [0.57–7.28]	104 (2.88) [0.99–8.14]
MB	17	15	2	16	14	2	16 (6.25) [1.11–28.33]	14 (7.14) [1.27–31.47]	2 (0.00)
ME	1	/	1	/	/	/	/	/	/
MM	56	2	54	53	2	51	52 (25.00) [15.23–38.21]	2 (50.00) [9.45–90.55]	50 (24.00) [14.30–37.41]
MM/B	7	2	5	3	1	2	2 (50.00) [9.45–90.55]	/	2 (50.00) [9.45–90.55]
MN	1	/	1	1	/	1	1 (0.00)	/	1 (0.00)
RF	86	45	41	78	40	38	77 (0.00)	39 (0.00)	38 (0.00)
Not determined	1	/	/	1	/	/	1 (0.00)	/	/
Total	392	174	217	363	158	204	350 (5.71) [3.73–8.66]	152 (2.63) [1.03–6.57]	197 (8.12) [5.06–12.78]

*ES Eptesicus serotinus*, *MS Miniopterus schreibersii, MB Myotis blythii*, *ME Myotis emarginatus*, *MM Myotis myotis*, *MM/B Myotis myotis/blythii*, *MN Myotis nattereri*, *RF Rhinolophus ferrumequinum*, *M* male, *F* female, *pos* positive

**Fig. 1:**
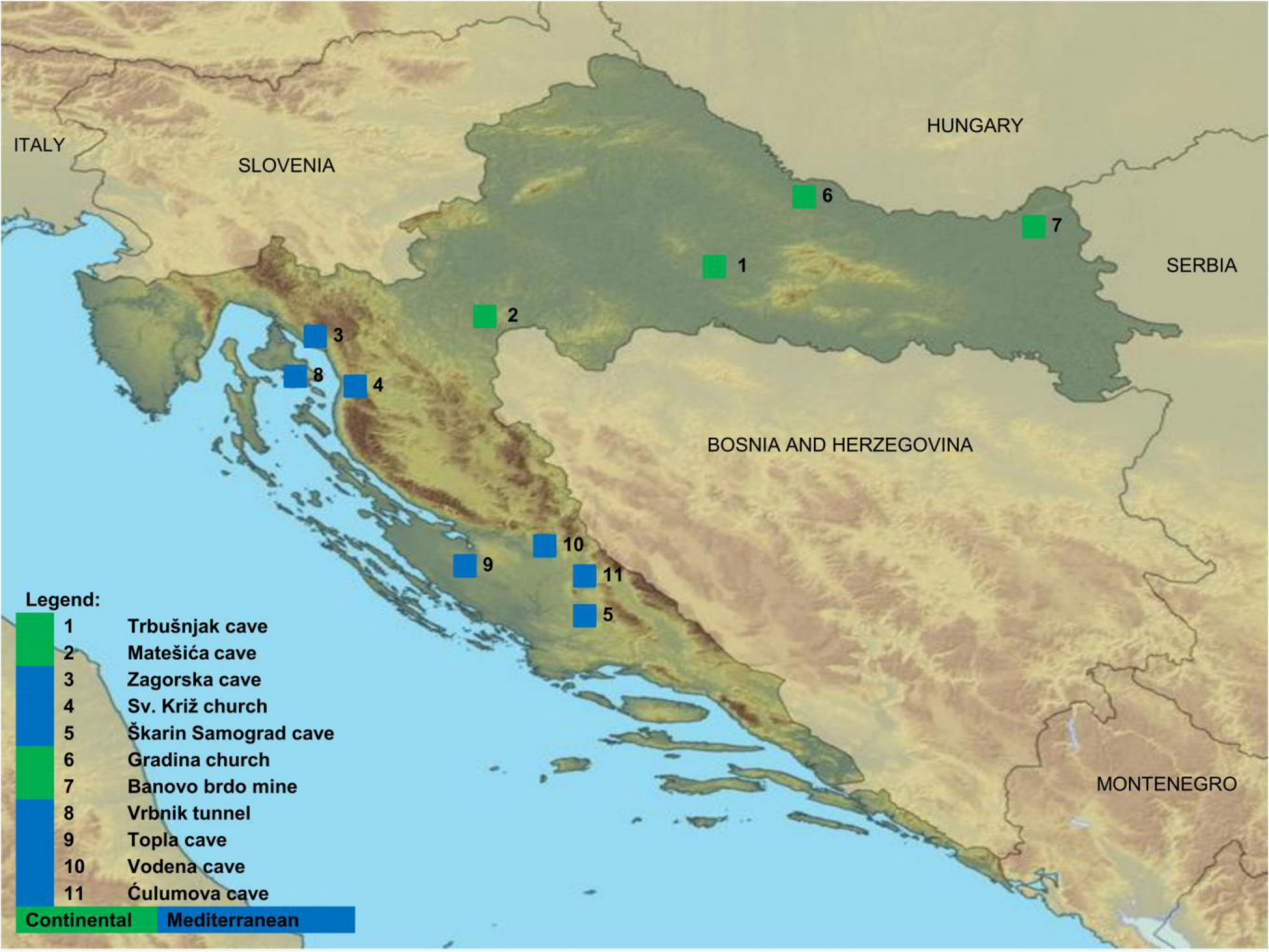
Locations of bat sampling in Continental (green) and Mediterranean (blue) Croatia. Source: https://hr.wikipedia.org/wiki/Datoteka:Croatia_map_blank.png

**Fig. 2:**
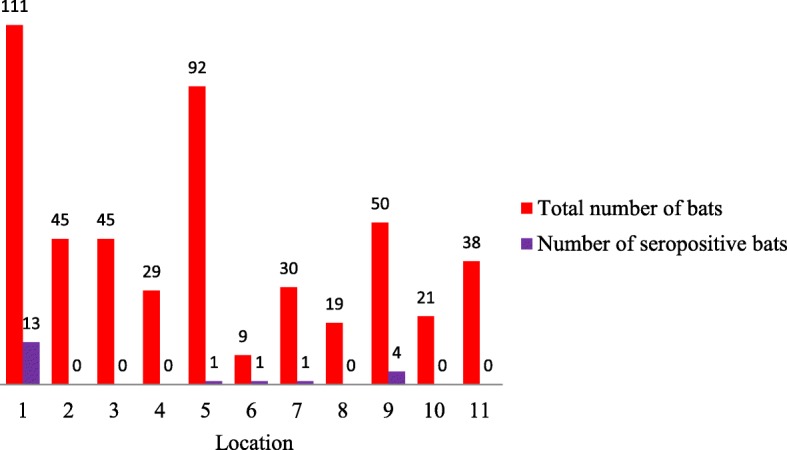
Total number of bats (red bars) and number of seropositive bats (purple bars) per location (1–11). Location designations are the same as on map (Fig. 1)

Overall, 195 samples were from four Continental locations, and 260 samples were from seven Mediterranean locations. Most of the samples were collected in location 1 (*n* = 111) and location 5 (*n* = 92; Figs. 1 and 2).

### Detection of EBLV-1 antibodies

In this study, 363 of 392 sampled bats were subjected to analysis (Table 2). Readable results were obtained for 350 animals. All samples were tested individually.

In total, 20 serum samples (range: 1.67–2.62 log D_50_, all ≥1:27) showed detectable levels of neutralizing antibodies against EBLV-1 from 16 females (*Myotis myotis/blythii, Myotis myotis, Miniopterus schreibersii*) and four males (*Myotis blythii, Myotis myotis*, *Miniopterus schreibersii*; Table 2). Seroprevalence among females was significantly higher than that among males (*p* < 0.001). Among seropositive bats, 65% belonged to *Myotis myotis* (13/20; Table 2), although the majority of bats caught in this survey were *Miniopterus schreibersii*. Seroprevalence was significantly higher in *Myotis myotis* than in *Miniopterus schreibersii* (*p* < 0.001).

Seropositive bats were found in five locations with the majority (15/20) found in Continental Croatia. At location 1 (Fig. 1.), where most bats were sampled (111/392), number of seropositive bats was the highest (13/392). At locations 9, four bats were seropositive, whereas at locations 5, 6 and 7 (Figs. 1 and 2) only one seropositive bat was found per site. In the remaining six locations, all bats were negative on the day of capture (locations 2–4, 8, 10, and 11; Figs. 1 and 2).

### Detection of lyssaviral RNA

All 453 oropharyngeal swabs were negative for the presence of lyssaviral RNA, suggesting that none of the bats were excreting virus in saliva at the time of sampling. Two samples were inappropriate for processing.

Beta-actin was detected in all the swabs analyzed (*n* = 453), indicating that host material was present on the swabs.

## Discussion

In Europe, due to the implementation of national rabies programs, which primarily focus on oral rabies vaccination (ORV) of wildlife, the numbers of rabies cases has dramatically decreased in non-flying mammals [15, 16]. However, distinct rabies epidemiological cycles occur in certain European bat species, and the public health impact of bat rabies in Europe should not be underestimated [17, 18]. Bats are the reservoirs for the majority of lyssavirus species, and available rabies vaccines do not confer efficient protection against all of these species. Additionally, minor bite wounds from small insectivorous bats could result in cryptic rabies, which is often reported in North America, although both EBLV-1 and EBLV-2 are less pathogenic than RABV [3].

In Croatia ORV was implemented in 2011, and the last case of rabies was reported in a fox in February 2014. Accordingly, we have focused our research on lyssaviruses circulating on autochthonous bats. An active surveillance program was undertaken to assess the potential public health risk and to elucidate lyssavirus epidemiology in Croatia and SEE.

Approximately 735 dead bats were submitted for rabies diagnosis from 1986 to 2017, and all were found negative by FAT and/or RT-PCR, consistent with reports from several European countries and dependent on number of samples tested [19–21]. Additionally, most tested bats were found dead by bat biologists and were in different stages of decomposition. Some carcasses were frozen for years before testing, or brain tissues were kept in ethanol [14], which could decrease possibility of detection of viral antigens by FAT and/or RT-PCR.

In this study, no lyssavirus RNA was detected in oropharyngeal swabs, suggesting that lyssavirus RNA was not being shed into the saliva of the bats sampled upon capture. These findings are consistent with previous studies conducted elsewhere in Europe [19, 22–24]. Similarly, intermittent excretion of virus in saliva was observed during experimental inoculations [25–27] and may explain the absence of lyssaviral RNA in oropharyngeal swabs.

In Germany, most RT-PCR positive results are associated with *E. serotinus* [20], whereas in Switzerland, most are associated with *Myotis daubentonii* [17]; these species are natural reservoirs for EBLV-1 and EBLV-2, respectively. The under-representation of *E. serotinus* and *Myotis daubentonii* in this study could also explain the absence of EBLV RNA. Although *E. serotinus* is a widespread species in Croatia [28], at the time of sampling, this species had abandoned roost at one location, and we did not have access to sample individuals from another location. In this study, we focused on anthropophilic and cave-dwelling bat species with known roosts; thus *Myotis daubentonii*, as a typical forest species, was not included [29]. Notably, Freuling et al. [25] emphasized that focusing on virus detection in live bats alone has limited effectiveness and should be accompanied by serological surveys.

In this study, for the first time, we confirmed the presence of anti-EBLV-1 antibodies among bats in Croatia and in SEE. Neutralizing antibodies were found in four bat species (*Miniopterus schreibersii, Myotis blythii, Myotis myotis, Myotis myotis/blythii*), with a seroprevalence of 5.71%. Although, for various reasons (challenge virus, test used, cut-off value), it is difficult to compare the results of serological testing between studies, similar seroprevalence rates were observed in Sweden [19], France [24], and Scotland [22].

In contrast, in neighboring countries (Serbia [30] and Slovenia [31, 32]) where active surveillance was conducted, neither virus neutralizing antibodies (VNA) nor virus was detected, although the number of investigated animals was similar to that in our study. In contrast, virus was detected in northern Hungary on a few occasions (*n* = 7) [21].

VNAs have been found in many bat species in several European countries; however, because of cross-reactivity, seropositivity cannot be linked to a specific lyssavirus [19]. In our study, few samples were positive for EBLV-1 but not tested for EBLV-2, RABV, or representatives of phylogroup III (such as LLEBV) due to the low volume of blood collected per bat. Although serological cross-reactivity between members of one phylogroup exists, higher sensitivity of the neutralization test is obtained when using host- specific EBLV-1 as the challenge virus [33]. Therefore, it is possible that we may have missed detection in some bat species because only one test virus was used particularly because *Miniopterus schreibersii* and *Myotis nattereri* have also been associated with WCBV and BBLV, respectively [3, 7]. Furthermore, LLEBV was detected in *Miniopterus schreibersii* found in Spain [34, 35] and recently in France (Picard-Meyer E, Beven V, Hirchaud E, Guillaume C, Larcher G, Robardet E, Servat A, Blanchard Y, Cliquet F: First Isolation of Lleida Bat Lyssavirus from a Schreiber’s bat in France, submitted).

*Miniopterus schreibersii* forms the largest winter and maternity colonies of all Croatian bat species [36] and was the most common bat sampled in this study. This species dwells in four of the five locations where seropositive bats have been found, and as seasonal migrators (> 350 km), they could be one of the dispersion vectors of the disease in Croatia and neighbouring countries [37, 38]. Record of *Miniopterus schreibersii* banded in Slovenia at location 1 confirms that this is possible [39].

However, only five seropositive *Miniopterus schreibersii* were found, and the bat species with the highest prevalence of VNA positivity was *Myotis myotis*. These findings are inconsistent with previous studies [24, 40], in which most records of VNA were found in *E. serotinus*. As described earlier, under-representation of *E. serotinus* in this study could explain these discrepancies. Detection of EBLV-1 VNA in 25% (13/52) of the analyzed *Myotis myotis* samples suggests that bats of this species were infected with EBLV-1 and may be also involved in dispersion of EBLV-1 in countries in SEE, such as Spain [37].

In our study, females were more frequently captured and were more prevalent among seropositive bats. This result could be a consequence of bat ecology and the time of sampling because the majority of sampling was performed in the spring, when maternity colonies consisting of pregnant females are formed. Additionally, pregnancy in bats during spring may change their immune responses with respect to lyssaviruses, which may have affected our capacity to determine detectable antibodies [22].

At location 1, a large number of positive samples (*n* = 13) was observed, likely because the most bats were sampled from this location (*n* = 111) over two consecutive years. This finding confirmed the importance of sampling more at every location and the need for prolonged monitoring of roosts. In this location in 2016, more positive bats were observed (*n* = 10) than in 2017 (*n* = 3). However, lack of previous data and unmarked bats prevented us from making conclusions related to the lyssavirus epidemiological cycle in that colony and emphasized the importance of bat ringing.

## Conclusions

In this study, we confirmed the presence of EBLV-1 antibodies in Croatia, suggesting the circulation of EBLV-1 in autochthonous bats, particularly in the continental part of the country. Although *E. serotinus* bats are thought to play a key role in the epidemiology of bat rabies in Europe [3], no conclusion have been made regarding their roles in bat rabies in Croatia.

Whether the lower seroprevalence of lyssaviruses will persist over time remains to be confirmed. Additionaly, testing of other resident bat species in Croatia should be performed, particularly for reservoir species, for species previously not sampled, and by using other lyssavirus species with mFAVN to assess the potential public health risks. All bat biologists should be aware of the risks and be vaccinated to prevent rabies transmission from bats. Education of the general public is strongly suggested, and any contact with bats should be considered a possible exposure.

## Methods

### Sample collection

In this study, we evaluated seven of 34 bat species present in the country, i.e., greater mouse-eared bat (*Myotis myotis*), lesser mouse-eared bat (*Myotis blythii*), Geoffroy’s bat (*Myotis emarginatus*), Schreiber’s bent-winged bat (*Miniopterus schreibersii*), greater horseshoe bat (*R. ferrumequinum*), serotine bat (*E. serotinus*), and Natterer’s bat (*Myotis nattereri*). Because of the morphological similarity between *Myotis myotis* and *Myotis blythii*, for 14 individuals, species could not be precisely determined. These individuals were designated *Myotis myotis/blythii* (Table 2).

Bats (Table 1) were captured in spring of 2016 and spring and autumn of 2017 at 11 locations in Continental **(***n* = 4) and Mediterranean (*n* = 7) Croatia (Figs. 1 and 2). From the selected locations, two were churches (locations 4 and 6), one was a tunnel (location 8), one was a closed mine (location 7), and seven were caves (locations 1–3, 5, 9–11). The locations were selected because they are important underground sites for bats in Croatia (churches excluded) [41], consistent with bat colony behaviors (anthropophilic or cave-dwelling bat species). Bat experts conducted captures using mist nets (Ecotone Mist Nets) at the entrances of caves during night (locations 2, 5, 7, and 10) or using hand nets inside colonies during the day (the other seven locations). In three locations (locations 1, 2, and 5), sampling was conducted repeatedly over two consecutive years. Since bats were not marked, recapture could not be assessed at these three locations.

During sampling, bats were placed individually in cotton bags and were identified by bat biologists according to morphological criteria [42]. Age, body mass, forearm length, sex, and reproductive status were recorded. Capturing, handling, and sampling of bats were approved by the State Institute for Nature Protection (UP/I-612-07/16–48/163).

Blood samples acquired from the uropatagial vein using a 26-G needle (BD Microlance, Becton, Dickinson &Co. Ltd., Drogheda, Ireland) were collected on small pieces of filter papers (Mini Trans-Blot; Bio-Rad, Hercules, CA, USA). A maximum of approximately 23 μl of blood was applied to each piece of filter paper, with the number of pieces varying between one and four per animal based on the size of the animal. Filter papers were dried in the laboratory and stored at − 20 °C until analysis.

Two oropharyngeal swabs were taken from each bat with a dry sterile swab (Copan Italia SpA, Brescia, Italy). One swab was preserved in 500 μL nucleic acid stabilization reagent (DNA/RNA Shield; Zymo Research, Irvine, CA, USA) for RT-PCR, and the second was preserved in 500 μL transport medium (Dulbecco’s modified Eagle’s medium [DMEM] supplemented with 10% fetal bovine serum [FBS] and 1% antibiotic / antimycotic) for further virus isolation in cases of positive RT-PCR results. The swabs remained in these solutions until processing, at which time the solution was aspirated and used in the assay. In the laboratory, swabs in DNA/RNA Shield were kept at room temperature, whereas swabs in the transport medium were stored at − 20 °C until testing.

After sampling, bats were offered glucose solution orally, and all were successfully released at the location of their capture.

Furthermore, at each location, we searched for potential bat cadavers, but none were found. Brains or other tissues from bats were not collected during this study.

### Sample analysis

#### Detection of anti-EBLV antibodies

Collected blood samples were tested for neutralizing anti-EBLV-1 antibodies with mFAVN tests. Samples soaked on filter papers were first diluted with growth medium, with 65 μL per piece of paper. The mFAVN test was performed according to a previously described protocol [43], except that the virus/ serum mix was distributed on 24- h old BHK-21 monolayers (1 × 10^5^ cells/mL) in 96-well plates (Thermo Fisher Scientific, Roskilde, Denmark). The challenge virus EBLV-1 was diluted at around 100 TCID_50_ per well. The complete growth medium used in the mFAVN test was DMEM (Sigma-Aldrich, St. Louis, MO, USA), supplemented with heat- inactivated FBS (10%; Gibco, US origin, Paisley, UK) and antibiotic / antimycotic (1%; Sigma-Aldrich). Microplates were incubated at 35 °C with 95% relative humidity and 5% CO_2_ for 48 h.

Owing to limited sample volume, samples were analyzed in duplicate to determine the presence of anti-EBLV-1 antibodies and serially diluted using a three-fold series. Because positive serum from an EBLV-1- infected bat was not available, a rabies immunoglobulin standard preparation (WHO International Laboratory for Biological Standards, Copenhagen, Denmark) was used as the positive control. FBS was used as negative control. Fluorescein isothiocyanate-conjugated anti-rabies virus monoclonal globulin (Fujirebio Diagnostics, Malvern, PA, USA), diluted according to the manufacturer’s instructions, was used as a conjugate. A reciprocal titer of 27 (1.67 log D_50_) was used as a positive cut-off [22, 24, 37].

#### Detection of lyssaviral RNA

All collected saliva samples were analyzed for the presence of beta-actin RNA and lyssavirus RNA by real -time and conventional RT-PCR, respectively.

Briefly, oropharyngeal swabs from 453 bats preserved in DNA/RNA Shield were vortexed and centrifuged at 3000 x g for 10 min. RNA was extracted from 230 μL supernatant samples using an iPrep PureLink Virus Kit (Invitrogen, Carlsbad, CA, USA) on an iPrep Purification Instrument according to the manufacturer’s instructions. RNA extracts were stored at − 20 °C until used.

To detect *Lyssavirus* RNA, hemi-nested RT-PCR was performed using a SuperScript III One-Step RT-PCR System with Platinum Taq DNA Polymerase (Invitrogen) according to a previously described protocol [44].

All amplifications were performed in a 2720 Thermal Cycler (Applied Biosystems, Foster City, CA, USA). PCR products were visualized under ultraviolet light after gel electrophoresis on 1.5% agarose. Positive (CVS) and negative (phosphate-buffered saline) controls were added for RNA extraction, RT-PCR, and hemi-nested PCR.

To prevent any false negative results due to the absence of oropharyngeal host material or degradation of RNA, a real – time TaqMan RT-PCR (qRT-PCR) was conducted on all samples using specific primers [45] targeting mammalian beta-actin. The qRT-PCR reaction was conducted using a Multiplex Real-Time One-Step RT-PCR Kit according to the manufacturer’s instructions (Qiagen, Hilden, Germany) and RotorGene Q (Qiagen).

### Statistical analysis

Comparison of sex and species distribution between seropositive bats were performed using non-parametric Wilcoxon Rank-Sum Tests. The 95% confidence intervals of seroprevalence data were calculated using STATA 13.1 (Stata Press, College Station, TX, USA). Results were considered significant when *p* values were less than 0.001.
